# 3D heterogeneous dose distributions for total body irradiation patients

**DOI:** 10.1120/jacmp.v12i3.3416

**Published:** 2011-06-01

**Authors:** Marie‐Claude Lavallée, Sylviane Aubin, Marie Larochelle, Isabelle Vallières, Luc Beaulieu

**Affiliations:** ^1^ Département de Radio‐Oncologie et Centre de Recherche en Cancérologie, CHUQ Pavillon L'Hôtel‐Dieu de Québec Québec PQ Canada G1R 2J6; ^2^ Département de Physique, de Génie Physique et d'Optique, Université Laval Québec PQ Canada G1K 7P4

**Keywords:** total body irradiation, treatment planning, dose distribution, translating couch, heterogeneities

## Abstract

One major objective of total body irradiation (TBI) treatments is to deliver a uniform dose in the entire body of the patient. Looking at 3D dose distributions for constant speed (*CstSpeed*) and variable speed (*VarSpeed*) translating couch TBI treatments, dose uniformity and the effect of body heterogeneities were evaluated. This study was based on retrospective dose calculations of 10 patients treated with a translating couch TBI technique. Dose distributions for *CstSpeed* and *VarSpeed* TBI treatments have been computed with Pinnacle^3^ treatment planning system in homogeneous (*Homo*) and heterogeneous (*Hetero*) dose calculation modes. A specific beam model was implemented in Pinnacle^3^ to allow an accurate dose calculation adapted for TBI special aspects. Better dose coverages were obtained with *Homo/VarSpeed* treatments compared to *Homo/CstSpeed* cases including smaller overdosage areas. Large differences between *CstSpeed* and *VarSpeed* dose calculations were observed in the brain, spleen, arms, legs, and lateral parts of the abdomen (differences between V100% mean values up to 57.5%). Results also showed that dose distributions for patients treated with *CstSpeed* TBI greatly depend on the patient morphology, especially for pediatric and overweight cases. Looking at heterogeneous dose calculations, underdosages (2%–5%) were found in high‐density regions (e.g., bones), while overdosages (5%–15%) were found in low‐density regions (e.g., lungs). Overall, *Homo/CstSpeed* and *Hetero/VarSpeed* dose distributions showed more hot spots than *Homo/VarSpeed* and were greatly dependent on patient anatomy. *CstSpeed* TBI treatments allow a simple optimization process but lead to less dose uniformity due to the patient anatomy. *VarSpeed* TBI treatments require more complex dose optimization, but lead to a better dose uniformity independent of the patient morphology. Finally, this study showed that heterogeneities should be considered in dose calculations in order to obtain a better optimization and, therefore, to improve dose uniformity.

PACS number: 87.55.D

## I. INTRODUCTION

Total body irradiation (TBI) is a particular radiotherapy treatment where the whole body of the patient needs to be irradiated. Since the target volume is too large to be irradiated with conventional fields, different techniques have been developed to overcome the related constraints. The main differences between these techniques reside in patient positioning, energy choice, dose rate, source‐to‐skin distance, and treatment field size.^(^ ^1^ ^–^ ^12^ ^)^ Clinical TBI studies are hard to implement since the number of eligible and comparable patients is small. In addition, pathologies, chemotherapy regimens, TBI techniques, and graft techniques also differ between patients. While it is hard to conduct clinical studies, publications have addressed some recommendations in order for physicists to be able to understand and compare the clinical outcomes.^(^ ^1^ ^,^ ^8^ ^)^ The following recommendations are common to most publications: dose uniformity in the target (entire body) should be within ± 10%, any relevant dose overdosages or underdosages greater than ± 5% should be recorded, and the maximum and minimum values should be well known.^(^ ^1^ ^,^ ^5^ ^,^ ^8^ ^)^ Therefore, everything that improves dose distribution knowledge should be promoted. Because TBI is a complex radiotherapy treatment, most institutions use very simple treatment planning approaches and a homogenous dose calculation. In order to increase uniformity, variable speed translating couch techniques were developed where couch velocities are optimized to provide a uniform dose in the whole body of the patient.^(^ ^8^ ^–^ ^13^ ^)^ In a previous study, the dose calculation accuracy of two treatment planning systems was explored.^(14)^ A dedicated TBI unit was commissioned in Pinnacle^3^ to allow an accurate dose calculation that takes into account the special aspects of the technique such as extended source‐to‐skin distance (SSD), large field, beam spoiler, and out of field dose contribution. In the literature, very few papers deal with TBI treatment planning issues, and none of them have studied the influence of the heterogeneities found in the entire body.

The scope of this study was to use the TBI specific beam commissioned in the Pinnacle^3^ treatment planning system to calculate dose distributions of TBI patients. This will allow the observation of the actual 3D dose distribution including regions where heterogeneities can be found, and the verification of the achieved dose uniformity in the entire volume of the body. This work focused on three different dose distribution calculation modes. The first one is a constant speed TBI treatment with a homogeneous dose calculation. The second and the third approaches are variable speed TBI treatments with homogeneous and heterogeneous dose calculations, respectively.

## II. MATERIALS AND METHODS

### A. TBI technique and treatment planning

The TBI technique studied is an AP/PA treatment where the patient lies on a table placed directly on the floor for a source‐to‐table distance of 214 cm.^(11)^ Treatments are delivered using a 6 MV photon beam and a field size of $14\times 34\;{\text{cm}}^{2}$ at isocenter. The dose rate is about 50 cGy/min at patient midplane. A beam spoiler made of acrylic (thickness of 1.3 cm) is hung on the linac head to increase the skin dose up to 95% of the prescribed dose. The prescription is 12 Gy in six fractions delivered over three days. This technique uses a variable speed translating couch, and the velocities are optimized to deliver a uniform dose at patient midplane along the craniocaudal midline axis. The treatment plan is based on two distinct CT scan sets: one for the AP and another for the PA part of the treatment. The planning dosimetry is done using a large number of beams (72 beams, size: $14\times 34\;{\text{cm}}^{2}$) equally distributed along the patient's craniocaudal midline in order to cover the entire length of the patient's body. This beam distribution was proven to properly simulate the movement of the treatment couch. Beam size and the number of beams could be modified to yield a better dose distribution, but dose computation time and optimization times would be prohibitive in the clinic. This technique is found to allow for a better than 4% dose uniformity at the patient midline while maintaining reasonable calculation times.^(11)^ Also, smaller beams in the craniocaudal direction would result in a higher number of speed transitions which are not smoothed in the current optimization algorithm. The resulting speed changes could be uncomfortable for the patient and potentially wear out the table motors. The dose normalization point is set at the umbilicus, as in other TBI techniques.^(^ ^1^ ^–^ ^3^ ^,^ ^15^ ^)^ Beam weight optimization (i.e., table speed optimization) is performed using dose calculation points located at the center of every CT slices. These dose values are normalized to the patient midplane dose value at the umbilicus. Field weights are optimized to provide a uniform dose at the patient's midline and are then converted to couch velocities. Both treatment parts (AP and PA) are optimized independently. Dose uniformity at the patient's midline was found to be better than 4%.^(11)^

In a previous study,^(14)^ treatment planning systems were explored to compare different dose calculation algorithms and to evaluate different beam commissioning approaches. The superposition‐convolution algorithm of the Pinnacle^3^ planning system (version 7.9u, Philips Medical Systems, Andover, MA) was found to provide an accurate dose calculation.^(16)^ The dose calculation accuracy was further improved by the commissioning of a TBI specific beam model, which was developed to account for the TBI irregular beam characteristics such as the extended source‐to‐skin distance, large field size, beam spoiler, and out of field dose contribution. It was found that the TBI specific beam model in Pinnacle^3^ provides a better than 2% dose calculation accuracy, except in the buildup region (surface to 1.5 cm depth) where differences between measurements and dose calculations reached 5%. The main restriction of this model is, therefore, related to shallow dose calculations.^(14)^

#### A.1 Patients and dose calculation

This study is based on retrospective dose distribution calculations of 10 patients treated with the aforementioned TBI technique. Patients were randomly selected for this study, and were of various sizes and heights including one pediatric and two overweight patients. Specifications about the length, the width at the umbilicus, and the thickness at the umbilicus of patients are summarized in Table 1.

**Table 1:** Description of the 10 patients: length of the patient body from head to toe in treatment position, width of the patient at the umbilicus, and thickness of the patient at the umbilicus.

*Patient* #	*Length (cm)*	*Width (cm)*	*Thickness (cm)*
1	181.0	37.6	24.6
2	177.2	31.6	19.9
3	176.0	35.6	21.1
4	160.9	28.1	15.8
5	185.2	32.0	20.0
6	174.0	28.5	15.3
7	174.0	28.1	15.3
8 (pediatric)	117.1	25.0	16.2
9 (overweight)	168.9	42.6	27.0
10 (overweight)	173.0	49.8	30.1

As previously mentioned, the treatment planning is based on the weight optimization of 72 beams covering the entire body of the patient to simulate the translating couch aspect of the treatment. As described in Section A above, since TBI AP and PA patient immobilization setups are different, the treatment planning is based on two distinct sets of CT images (AP and PA), and treatment dose optimizations are performed separately. In this study, in order to evaluate the actual 3D dose distribution in the entire body of the patient, the treatment plan optimization was performed on only one CT exam (AP), with both AP and PA fields calculated and optimized with this set of images. Since AP and PA treatment positions are slightly different, the real treatment plan cannot be optimized using only a single set of CT images, but this approximation is adequate for the present study in order to evaluate 3D dose distributions. Dose distributions were first computed in a homogeneous dose calculation mode with equal field weights to simulate a constant couch speed. These computed distributions were kept and referred to as the homogeneous constant couch speed techniques (*Homo/CstSpeed*). In this instance, the dose perturbations arising from the heterogeneities in the body are neglected. In a second step, field weights were optimized with a uniformity optimization objective set to 2%. Optimized field weights were then converted into velocities used to deliver the variable speed translating couch TBI treatment. This homogeneous and optimized dose distribution (*Homo/VarSpeed*) does not take into account heterogeneities, but it represents the current method used to plan clinical TBI treatments. In a final step, the *Homo/VarSpeed* plans were recalculated (but not reoptimized) using a heterogeneous dose calculation mode (*Hetero/VarSpeed*) in order to include body heterogeneities. No heterogeneous dose calculation optimization was performed, since the actual optimization technique is based on dose point instead of dose volume calculations. For this type of optimization, dose calculation point locations are critical. For instance, if the calculation point on the CT slice is placed close to or inside a localized heterogeneity (e.g., within the spine) and if this heterogeneity is not representative of the entire CT slice, this could lead to an error in the optimization process.

For every patient, the whole body, brain, heart, kidneys, liver, lungs, bone marrow, and spleen were contoured in order to create dose volume histograms (DVH) and to study the 3D dose volume distributions. During treatments, lead attenuators are used to decrease the dose to organs at risk. Attenuator design and dose calculation represent challenges in moving to TBI techniques.^(^ ^10^ ^,^ ^17^ ^,^ ^18^ ^)^ In a previous study, dose distributions under the attenuator were characterized, and design recommendations were proposed to overcome the lack of dose coverage related to translating couch TBI treatments.^(18)^ Because attenuator designs and protected organs vary between practices, it was decided to not include them in this work.

## III. RESULTS

The dose distributions of 10 patients were compared. Dose uniformity in the whole body was evaluated for every case studied and was represented by the V105%, V100%, and V95% parameters, which are the fraction of the volume that received 105%, 100%, and 95% of the prescribed dose, respectively. Results are presented in Table 2. Looking at these results, not much differences are observed between the V95% and V100% mean values, but V105% mean values of *Homo/CstSpeed* (mean=26.5%) and *Hetero/VarSpeed* (mean=17.8%) were both higher than the V105% mean values of *Homo/VarSpeed* (mean=11.0%). Results also showed higher standard deviation values (σ) for *Homo/CstSpeed* (between 7.5 and 21.5), compared to *Hetero/VarSpeed* (between 4.0 and 12.8) and to *Homo/VarSpeed* (between 3.6 and 10.6).

**Table 2:** Mean V95%, V100%, and V105% values (%) and associated standard deviations (σ) in parenthesis for the whole body. Values were found for Homo/CstSpeed, Homo/VarSpeed, and Hetero/VarSpeed.

	*Homo/CstSpeed (%)*	*Homo/VarSpeed (%)*	*Hetero/VarSpeed (%)*
V95%	81.3 (7.5)	84.9 (3.6)	84.2 (4.0)
V100%	55.2 (20.5)	54.1 (10.6)	58.0 (12.8)
V105%	26.5 (21.5)	11.0 (5.4)	17.8 (9.6)

Specific regions of the body were also evaluated, and results are presented in Tables 3and 4, in which are reported the V100% and V95% values for the patients' whole body, brain, lungs, kidneys, liver, bone marrow, and spleen. It was observed that the V100% and V95% mean values were significantly smaller for *Homo/CstSpeed* than for *Homo/VarSpeed*, especially for the brain and the spleen where mean values were 21.8% and 57.5% for *Homo/CstSpeed* and 79.3% and 90.2% for *Homo/VarSpeed*, respectively. In comparing *Homo/VarSpeed* and *Hetero/ VarSpeed* results, high differences between mean V100% values were found (up to 42.7%), except for the entire body and the spleen for which the differences were smaller. V95% mean values of all organs were more similar, except for the brain and lungs where higher differences of 13.7% and 6.1%, respectively, were observed.

**Table 3:** Mean V100% values (%) and associated standard deviations (σ) in parenthesis for different regions of the body. Values were found for Homo/CstSpeed, Homo/VarSpeed, and Hetero/VarSpeed.

*Body Parts*	*Homo/CstSpeed (%)*	*Homo/VarSpeed (%)*	*Hetero/VarSpeed (%)*
Body	55.2 (20.5)	54.1 (10.6)	58.0 (12.8)
Brain	21.8 (39.9)	79.3 (11.5)	36.6 (17.9)
Lungs	54.0 (42.4)	41.3 (27.0)	83.0 (31.0)
Left Kidney	39.9 (44.4)	55.1 (31.4)	71.0 (29.8)
Right Kidney	44.0 (42.2)	60.8 (30.9)	73.2 (27.3)
Liver	44.9 (45.4)	69.2 (19.8)	55.6 (25.5)
Bone Marrow	49.4 (36.2)	67.2 (17.1)	49.0 (28.4)
Spleen	57.5 (45.0)	90.2 (13.6)	89.7 (11.8)

**Table 4:** Mean V95% values (%) and associated standard deviations (σ) in parenthesis for different regions of the body. Values were found for Homo/CstSpeed, Homo/VarSpeed, and Hetero/VarSpeed.

*Body Parts*	*Homo/CstSpeed (%)*	*Homo/VarSpeed (%)*	*Hetero/VarSpeed (%)*
Body	81.3 (7.5)	84.9 (3.6)	84.2 (4.0)
Brain	43.9 (40.8)	99.9 (0.1)	86.2 (27.5)
Lungs	90.1 (18.0)	94.6 (13.5)	88.5 (30.3)
Left Kidney	94.2 (16.2)	97.6 (7.6)	99.7 (0.8)
Right Kidney	96.3 (8.1)	100.0 (0.0)	99.6 (1.3)
Liver	88.9 (19.9)	100.0 (0.0)	94.1 (18.4)
Bone Marrow	92.7 (10.3)	100.0 (0.1)	94.9 (16.0)
Spleen	93.4 (13.0)	99.0 (3.2)	100.0 (0.0)

**Figure 1:**
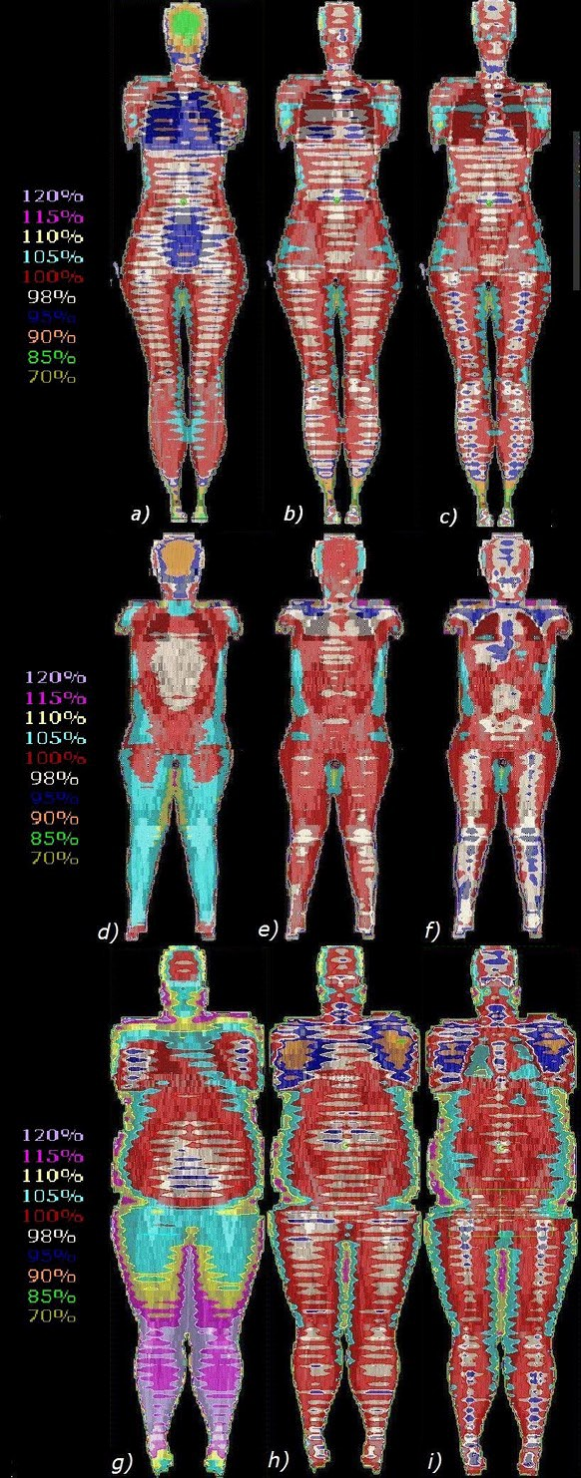
Coronal dose distributions of an average patient (first row), a pediatric case (second row), and an overweight patient (last row) calculated for *Homo/CstSpeed* (left column), *Homo/VarSpeed* (middle column), and *Hetero/VarSpeed* (right column).

**Figure 2:**
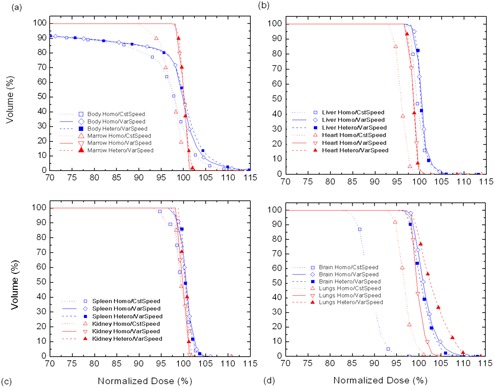
Dose volume histograms of an average patient. *Homo/CstSpeed*, *Homo/VarSpeed*, and *Hetero/VarSpeed* for: (a) body and bone marrow, (b) liver and heart, (c) kidney and spleen, and (d) brain and lungs.

Examples of average dose distributions at midplane are displayed in Fig. 1. This figure illustrates the dose distributions of a constant speed treatment with homogeneous dose calculation (*Homo/CstSpeed*, first column), a variable speed treatment with a homogeneous dose calculation (*Homo/VarSpeed*, second column), and a variable speed treatment calculated with a heterogeneous dose calculation (*Hetero/VarSpeed*, third column) for an average patient (first row), a pediatric case (second row), and an overweight case (third row). Figure 2 records examples of dose volume histograms of the average patient presented in (Figs. 1a), (b), and (c). Except for the two overweight cases, all the *Homo/CstSpeed* patients showed systematic underdosages of the brain ranging from 3% to 15% of the prescribed dose (see (Figs. 1a) and (d)). For all cases, overdosages between 5% and 10% were observed to the arms, legs, and each sides of the abdomen. The overdosage areas were bigger in most of the *Homo/CstSpeed* and *Hetero/VarSpeed* computation modes. For the pediatric case, it was observed that the legs received a greater dose in the constant speed treatment compared to the variable speed treatment, resulting in important overdosages of 5% ((Figs. 1d), (e), and (f)). For the overweight patients, results for *Homo/CstSpeed* (Fig. 1(g) showed important overdosages of 10% to 20% of the prescribed dose in the legs. Overdosed areas were significantly higher for overweight patients in all dose computation modes, with values between 5% and 30%, and they were located mainly in the lateral sides of the abdomen as well as the arms and legs. Looking at heterogeneous dose distributions ((Figs. 1c), (f), (i), and Fig. 2), radiation underdosages of about 2% to 5% were observed in bony regions, while important overdosages of about 5% to 15% were observed in air regions such as lungs.

## IV. DISCUSSION

### A. Constant speed vs. variable speed TBI treatments

The first part of this study has shown the degree of dose uniformity obtained using two different TBI treatment techniques: constant and variable speed translating couch. The main difference between constant speed and variable speed treatment plans are the number of optimization parameters. The constant speed treatment uses a one‐dimensional optimization based on the dose calculation at a point placed at the midplane and aligned with the umbilicus to find one velocity used for the entire treatment. Because there is only one parameter to determine, the main advantage resides in treatment planning simplicity. On the other hand, dose uniformity greatly depends on the patient anatomy itself and, therefore, dose uniformity is expected to be better for patient with a relatively uniform body thickness. The variable speed treatment requires a two‐dimensional treatment planning optimization process, where multiple dose points are placed at the center of every CT slice along the craniocaudal axis for a 2D optimization leading to a variable speed profile. In this case, since the craniocaudal direction is optimized, the dose uniformity is better than the one obtained with the constant speed treatment.

When comparing *Homo/CstSpeed* and *Homo/VarSpeed* results, not many differences were observed between V95% and V100% values of the entire body, but overdosages were higher in *Homo/CstSpeed* cases (see Table 2). Looking at the results associated with different regions of the body (Tables 3and 4), it was observed that V100% and V95% values are significantly smaller for *Homo/CstSpeed* than for *Homo/VarSpeed* dose calculations, especially for the brain and spleen where differences between V100% mean values were 57.5% and 35.4%, respectively. Higher V100% and V95% values are associated with better dose coverage. Results also showed that standard deviation values were smaller for *Homo/VarSpeed* than for *Homo/CstSpeed*, which means that the dose distributions for patients treated with variable speed treatments are more similar. On the other hand, greater standard deviation values found for *Homo/CstSpeed* indicated that dose distributions in the entire body, as well as in every region of the body, were greatly dependent on patient morphologies.

Midplane coronal *Homo/CstSpeed* dose distributions of the average and pediatric cases ((Figs. 1a), (d), and Fig. 2(d) showed systematic underdosages of the brain (3% to 15%). This is related to patient morphology and the lack of photon scatter around the head region. Underdosages of the brain were not found in variable speed treatments because the lack of photon scatter was taken into account during the couch velocity optimization process. For the two overweight patients (e.g., Fig. 1(g), brains were mostly covered by 98% to 100% isodoses in the *Homo/CstSpeed.* This is explained by the choice of velocity based on the patient thickness at the umbilicus which leads to a slower velocity and, therefore, an increased dose to the brain. For the pediatric patient, it was observed that the legs received greater doses in the constant speed treatment (Fig. 1(d) compared to the variable speed treatment (Fig. 1(e). Only one pediatric case was included in this study and, as a result, no comparison could be made with another similar case. Since the velocity in the constant speed treatment is calculated from the patient thickness at the umbilicus, the dose distribution is strongly dependent on patient morphology, and leg overdosage was probably related to this effect. For overweight patients (Fig. 1(g), constant speed treatment led to a less‐uniform dose compared to the uniformity obtained for average patients with important overdosages of 10% to 30% in legs, arms, and lateral sides of the abdomen; this is also related to patient morphology and the treatment velocity choice. Finally, overdosage areas of 5% to 10% located on lateral parts of patient bodies were observed in both constant speed and variable speed cases, but they were bigger in most of the *Homo/CstSpeed* treatments. Therefore, even if in the variable speed treatment the dose along the midplane craniocaudal line seemed uniform, the distribution is less uniform when going away from the central line. This is mainly due to the fact that dose modulation is performed by the couch speed variation and is, therefore, limited to the longitudinal direction. Given the nonuniform densities and thicknesses in the patient along the lateral direction, which are not taken into account during speed optimization, overdosages in the body sides will occur.

### B. Homogeneous vs. heterogeneous dose calculations

An accurate 3D heterogeneous dose calculation (as offered by Pinnacle^3^) allows the visualization of realistic dose distributions in the whole body of TBI patients. To keep the treatment planning as simple as possible, most TBI techniques do not take into account the heterogeneities of the body. The objective of the second part of the study was to look at heterogeneous dose distributions and compare them to the related homogeneous distributions. Regions of high‐density heterogeneities will attenuate the dose, while regions of low density will create overdosed areas. When comparing *Homo/VarSpeed* and *Hetero/VarSpeed* results (presented in Table 2), not a great deal of differences were observed between V95% and V100% values of the entire body, but overdosages (V105% values) were higher for *Hetero/VarSpeed* cases. Looking at the values associated with different regions of the body as presented in Tables 3 and 4, it was observed that V100% and V95% values were different for *Homo/VarSpeed* and for *Hetero/VarSpeed* dose calculations, especially for the V100% values (differences up to 42.7%). Standard deviation values for different organs, except for kidneys and spleen, were also greater for *Hetero/VarSpeed* than for *Homo/VarSpeed*. This means that dose coverage of these organs was influenced by body heterogeneities and, in reality, more hot spots were found with heterogeneous dose calculation mode.

Heterogeneous dose distributions for variable speed treatments ((Figs. 1c), (f), (i), and Fig. 2) showed radiation underdosages of about 2% to 5% in bone regions, and overdosages of about 5% to 15% in low‐density regions as lungs. The largest differences between the homogeneous (Fig. 1(b), (e), (h), and Fig. 2(d) and heterogeneous ((Figs. 1c), (f), (i), and Fig. 2(d) dose distributions were found in lungs. It is important to keep in mind that attenuators are used during the treatment to decrease dose to the organs at risk. Lungs are the most frequently protected ones. As attenuators are meant to decrease the dose, it can be expected that observed radiation overdosage effects will be smaller. Finally, as observed for *Homo/CstSpeed* and for *Homo/ VarSpeed*, results for *Hetero/VarSpeed* showed that the dose is less uniform when going away from the central craniocaudal line. Overdosed areas of 5% to 20% of the prescribed dose were also found in the lateral sides of abdomen, arms, and legs.

In this study, the energy used for TBI is 6 MV (our clinical standard) and the TBI‐specific beam model in Pinnacle^3^ was developed for that specific energy. Therefore, no results were obtained for higher photon energies, but effects of body heterogeneities on dose distributions are expected to be smaller. However, the method we developed for extended SSD techniques beam model is applicable to any beam energy without loss of generality.^(14)^

## V. CONCLUSIONS

This study has shown the degree of uniformity obtained with constant and variable speed translating couch TBI techniques. It was observed that constant speed TBI treatments based on a single point dose calculation led to a nonuniform dose distribution with underdosed and overdosed areas strongly related to patient morphology. With its two degrees of freedom, the variable speed TBI treatment optimization provides a more uniform dose in the whole body, especially at the patient midplane and midline along the craniocaudal axis. While the dose is uniform on the central axis of the patient, overdosages are still found in the lateral parts of the abdomen, upper legs, and arms. In a second step, the effects of body heterogeneities on dose distributions were explored for variable speed TBI treatments. It was observed that overdosages and underdosages were found in low‐density regions (e.g., lungs) and in high‐density regions (e.g., bones), respectively. To achieve optimal dose uniformity in translating couch TBI technique, a treatment planning optimization should be done on volumes instead of on dose points and in the heterogeneous dose calculation mode. Since most institutions use very simple dose calculation processes for TBI, it is likely that deviations in dose uniformity of ± 10% or even more are to be expected. These deviations in dose distribution will depend on the optimization process and the heterogeneities, as well as patient morphology, especially for those who have nonuniform body thicknesses.

## ACKNOWLEDGMENTS

This research was supported by the Natural Sciences and Engineering Research Council (NSERC) grant # 262105. We acknowledge Philips Medical Physics Systems for providing the latest version of the Pinnacle^3^ treatment planning research software. The authors would like to thank our colleague Ghyslain Leclerc for useful comments on the manuscript.
